# Introduction of Inactivated Polio Vaccine, Withdrawal of Type 2 Oral Polio Vaccine, and Routine Immunization Strengthening in the Eastern Mediterranean Region

**DOI:** 10.1093/infdis/jix133

**Published:** 2017-07-01

**Authors:** Kamal Fahmy, Lee M. Hampton, Houda Langar, Manish Patel, Tahir Mir, Chandrasegarar Soloman, Andreas Hasman, Nasir Yusuf, Nadia Teleb

**Affiliations:** 1 Eastern Mediterranean Region Office, World Health Organization, Cairo, Egypt;; 2 Global Immunization Division, Centers for Disease Control and Prevention, Atlanta, and; 3 Task Force for Global Health, Decatur, Georgia;; 4 Middle East and North Africa Regional Office, United Nations Children’s Fund (UNICEF), Amman, Jordan;; 5 South Asian Regional Office, UNICEF Kathmandu, Nepal; and; 6 East and South Africa Regional Office, UNICEF Nairobi, Kenya

**Keywords:** inactivated polio vaccine, routine immunization strengthening, tOPV-bOPV, switch (“the switch”)

## Abstract

The Global Polio Eradication Initiative has reduced the global incidence of polio by 99% and the number of countries with endemic polio from 125 to 3 countries. The Polio Eradication and Endgame Strategic Plan 2013–2018 (Endgame Plan) was developed to end polio disease. Key elements of the endgame plan include strengthening immunization systems using polio assets, introducing inactivated polio vaccine (IPV), and replacing trivalent oral polio vaccine with bivalent oral polio vaccine (“the switch”). Although coverage in the Eastern Mediterranean Region (EMR) with the third dose of a vaccine containing diphtheria, tetanus, and pertussis antigens (DTP3) was ≥90% in 14 countries in 2015, DTP3 coverage in EMR dropped from 86% in 2010 to 80% in 2015 due to civil disorder in multiple countries. To strengthen their immunization systems, Pakistan, Afghanistan, and Somalia developed draft plans to integrate Polio Eradication Initiative assets, staff, structure, and activities with their Expanded Programmes on Immunization, particularly in high-risk districts and regions. Between 2014 and 2016, 11 EMR countries introduced IPV in their routine immunization program, including all of the countries at highest risk for polio transmission (Afghanistan, Pakistan, Somalia, and Yemen). As a result, by the end of 2016 all EMR countries were using IPV except Egypt, where introduction of IPV was delayed by a global shortage. The switch was successfully implemented in EMR due to the motivation, engagement, and cooperation of immunization staff and decision makers across all national levels. Moreover, the switch succeeded because of the ability of even the immunization systems operating under hardship conditions of conflict to absorb the switch activities.

Since its launch in 1988, the Global Polio Eradication Initiative (GPEI) has reduced the global incidence of polio by 99% and the number of countries with endemic polio from 125 to 3 countries, as of 15 August 2016 [1]. In May 2012, the World Health Assembly called on the World Health Organization (WHO) to develop and finalize a comprehensive poliomyelitis endgame strategy. The Polio Eradication and Endgame Strategic Plan 2013–2018 (Endgame Plan) was developed to end polio disease from both wild poliovirus and circulating vaccine-derived polioviruses (cVDPVs) [2]. Countries of the Eastern Mediterranean Region (EMR) of WHO have made marked progress against polio eradication, with only Pakistan and Afghanistan continuing to have endemic polio [3]. From its beginning, the regional effort against polio has depended on use of oral poliovirus vaccine (OPV), most commonly trivalent OPV (tOPV). However, although OPV is very effective in preventing poliovirus infections as well as paralytic poliomyelitis, the attenuated poliovirus in OPV can undergo genetic changes during replication, and rarely, in communities with low vaccination coverage, can result in vaccine-derived polioviruses (VDPVs) capable of causing paralytic polio [4].

The EMR has been affected by multiple outbreaks of polio caused by cVDPVs, including type 2 cVDPV (cVDPV2) outbreaks in Egypt, Somalia, Afghanistan, Yemen, and Pakistan and cVDPV3 outbreaks in Yemen [5, 6]. In total, the EMR has had at least 164 cases of paralytic polio caused by cVDPV2s and 4 cases of paralytic polio caused by cVDPV3s since 1988. The vast majority of those cases have been identified since 2006. At the same time, no cases of polio caused by wild poliovirus type 2 have been detected anywhere in the world since 1999 [7]. In 2009, bivalent oral polio vaccine (bOPV), which contains only attenuated types 1 and 3 polioviruses, became available [8]. In addition to not carrying the risk of causing cVDPV2s, bOPV generates better immunogenicity against types 1 and 3 polioviruses [8]; therefore, the EMR along with the rest of the world decided in 2012 to begin preparations for ceasing tOPV use and switching to bOPV use [2].

Switching from tOPV to bOPV is not without risks. If tOPV use stopped while cVDPV2 transmission was still ongoing, an outbreak might occur or worsen [9]. The existence of civil unrest in multiple countries in the EMR and the resulting disruptions in immunization services, as well as low population immunity to polio infections, required implementation of a multilayered effort to minimize the risks involved with ceasing tOPV use [10, 11].

The global and regional plans for switching from tOPV to bOPV contained multiple elements for minimizing and mitigating the inherent risks [12]. To provide protection against paralytic polio caused by cVPDV2s as well as provide some protection against type 2 poliovirus infections, all countries not already using inactivated polio vaccine (IPV), which cannot cause cVDPV2s, were advised to introduce IPV into their immunization schedules. To reduce the risk for spread of type 2 polioviruses from countries that continued to use tOPV to other countries that had ceased tOPV use, a globally synchronized switch from tOPV to bOPV was planned for April 2016, timed in the low poliovirus transmission season. To further boost population immunity to type 2 poliovirus infections, mass vaccination campaigns with tOPV were held in many high-risk countries before the switch. To guard against accidental use of tOPV after the switch from tOPV to bOPV, all countries engaged in an extensive monitoring process to check cold chain stores and health facilities to ensure that no tOPV was stored in the cold chain after the switch.

The objective of this article is to reflect the activities related to the implementation of the Endgame Plan in the EMR in light of the serious challenges faced by some EMR countries, particularly the activities of the WHO Eastern Mediterranean Regional Office (WHO EMRO) and the United Nations Children’s Fund (UNICEF) Middle East and North Africa Regional Office (UNICEF MENA), Eastern and Southern Africa Regional Office, and Regional Office for South Asia.

## ROUTINE IMMUNIZATION IN THE EASTERN MEDITERRANEAN REGION

In line with the Global Vaccine Action Plan [13], the EMR aimed at achieving by 2020 at least 90% routine immunization (RI) coverage nationally and 80% in every district, for all vaccines provided through the national Expanded Programme on Immunization (EPI).

**Figure 1.**
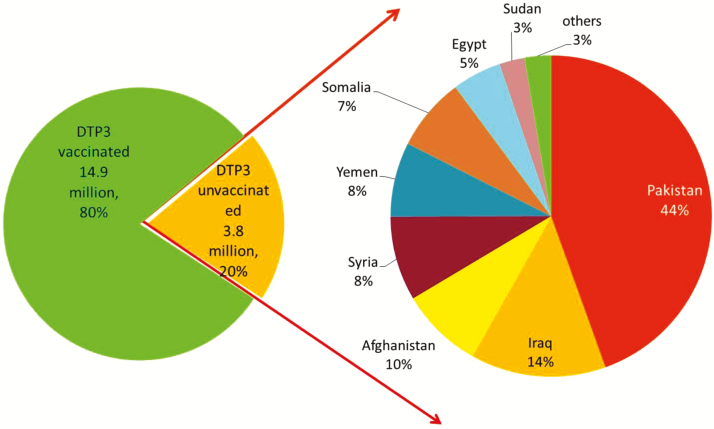
World Health Organization/United Nations Children’s Fund (UNICEF) estimates of proportion of children who had received 3 doses of diphtheria-tetanus-pertussis–containing vaccine by 1 year of age in 2015 in the Eastern Mediterranean region. Source: www.who.int/immunization_monitoring/globalsummary/timeseries/tswucoveragedtp3.html. World Health Organization and UNICEF national immunization coverage estimates, 2016. Abbreviations: OPT, Occupied Palestinian Territory; UAE, United Arab Emirates.

**Figure 2.**
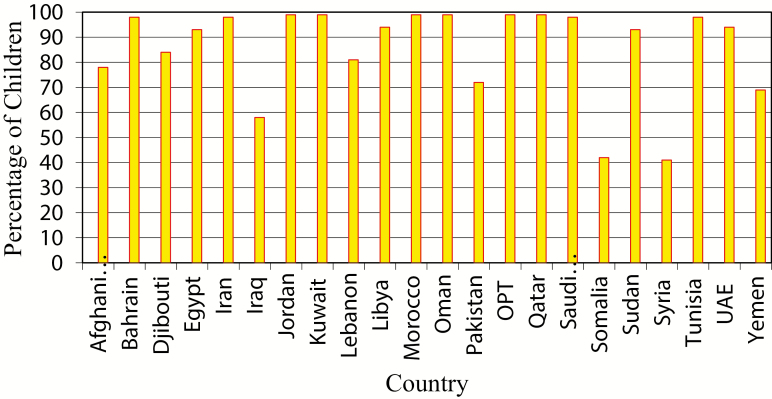
Distribution of unvaccinated children in the Eastern Mediterranean region by country, 2015. Source: www.who.int/immunization_monitoring/globalsummary/countries?countrycriteria%5Bcountry%5D%5B%5D=AFG&commit=OK. World Health Organization/United Nations Children’s Fund national immunization coverage estimates, 2016. Abbreviation: DTP3, third dose of a vaccine containing diphtheria, tetanus, and pertussis antigens.

EMR countries have achieved remarkable improvement in routine vaccination coverage during the past 2 decades. Coverage with the third dose of a vaccine containing diphtheria, tetanus, and pertussis antigens (DTP3) was >90% in 14 countries in 2015 based on WHO and UNICEF estimates (Figure 1), and the regional average of the regional DTP3 coverage reached 86% in 2010 based on WHO/UNICEF estimates. However, with the prevailing geopolitical situation and conflicts in the region, DTP3 coverage in the EMR dropped from 86% in 2010 to 80% in 2015, and 3.8 million infants missed receiving their third dose of DTP vaccine in the same year; most of these infants were in 6 countries of the region (Figure 2). Overall regional coverage of the third dose of OPV (OPV3) in the EMR similarly decreased from 86% in 2010 to 80% in 2015, but with vast intercountry and intracountry variations. OPV3 coverage was 90%–99% in 14 countries during 2010–2015. During this period, OPV3 coverage increased from 73% to 75% in Lebanon, and from 47% to 49% in Somalia, but decreased in Syria from 83% to 50%.

## STRENGTHENING RI THROUGH INTEGRATED EPI AND GPEI ACTIVITIES

A key activity of the Polio Endgame Plan focuses on strengthening RI using polio assets in 10 focus countries with the largest polio assets and weak immunization systems. Three of these countries are in the EMR: Pakistan, Afghanistan, and Somalia. Each of these countries developed a draft plan (“ONE EPI Plan”) and put forth efforts to integrate national EPI and GPEI activities by using GPEI-funded assets, staff, structure, and activities to strengthen the immunization system, particularly in high-risk districts and regions.

### Pakistan

Pakistan developed an EPI-GPEI integration plan in 2013. The initial plan included 16 pilot districts, later expanded to an additional 30 districts, including districts in the Federally Administered Tribal Areas. Within the 30 additional districts, polio staff such as the District Polio Eradication Officers and Union Council Medical Officers received training on RI support. These staff subsequently provided aid in RI microplanning, monitoring, and reporting for district and provincial meetings. Polio social mobilizers were also trained to promote RI and to report and refer unvaccinated children to immunization services. Because of the decentralized government health structure in Pakistan, the national EPI plan was developed separately from the 8 specific plans for each of the provinces.

### Afghanistan

Afghanistan implemented a pilot project using polio assets to strengthen RI in 30 districts (6 of which were low-performing districts in the east and south) with the aim of achieving a 10% annual reduction in the number of unimmunized children from 2014 through 2018. Within the 30 pilot districts, polio officers were trained in RI, assisting with microplanning, evaluating, and monitoring 30% of fixed and outreach vaccination sessions, and supporting vaccine-preventable diseases and acute flaccid paralysis surveillance. Social mobilizers were trained to promote RI and to identify, trace, and refer unvaccinated children. Polio field staff also attended weekly EPI management meetings to coordinate actions and share findings.

### Somalia

Polio staff and equipment in Somalia have been very useful for providing RI services in conflict regions because of an ongoing civil war concentrated in the South Central Zone. Throughout Somalia, polio staff were closely involved in the RI system, including collection, transmission, and analysis of data as well as vaccine management and monthly program reviews. Using GPEI resources, the federal government of Somalia has been improving RI implementation and coverage in high-risk areas not reached by the current RI program in the Banadir region of Somalia’s South Central Zone and the Marrodjex and Sool regions of Somaliland. For example, all GPEI staff in Somalia employed by WHO and UNICEF have received training to assist with RI activities, including a detailed microplanning workshop for maternal and child health leads and workshops for community health workers in conflict zone. Despite the ongoing conflicts, Somalia has developed an updated integrated EPI/GPEI plan that includes specific activities for strengthening of RI using GPEI-funded workers and assets.

## INACTIVATED POLIOVIRUS VACCINE INTRODUCTION IN ROUTINE IMMUNIZATION

In alignment with the Endgame Plan, the WHO’s Strategic Advisory Group of Experts (SAGE) on Immunization recommended in 2013 that all countries using only OPV should add at least 1 dose of IPV to their national immunization schedule [14]. While the addition of IPV to RI programs involves similar planning and implementation process as for other new vaccine introductions [15], several factors relevant to the introduction of IPV in the EMR warrant consideration. These include the unprecedented strict timelines so that the introduction would occur before the switch, financial support for the accelerated strategy, complexities of multiple injections and possible health workers’ and caregivers’ aversion to it, global supply shortages, and communication nuances related to IPV.

## ACCELERATED IPV INTRODUCTION IN THE EASTERN MEDITERRANEAN REGION

IPV was included in the vaccination schedule of 10 of the EMR’s 22 (45%) countries before the implementation of the Endgame Plan. Of the 11 countries that had to introduce IPV into the RI program, 4 were deemed by GPEI to be at high risk of cVPDV2 outbreak or importation [16].

During 2014–2016, 11 countries introduced IPV in their RI program (Table 1), including all of the high-risk countries (Afghanistan, Pakistan, Somalia, and Yemen). Iraq and Libya introduced IPV as a combination vaccine (hexavalent vaccine) whereas others introduced standalone IPV. All of these introductions were nationwide on the same day, except in Pakistan, which chose a phased introduction over a few months beginning in July 2015 because of its decentralized health structure.

**Table 1.** Dates of Inactivated Polio Vaccine Introduction in the Eastern Mediterranean Region, 2014–2016

Country	Planned IPV Introduction Date	Actual IPV Introduction Date
Afghanistan	June 2015	September 2015
Djibouti	October 2015	April 2016
Egypt	December 2015	Not yet introduced
Iran	August 2015	September 2015
Iraq	July 2015	January 2016
Libya	April 2014	April 2014
Morocco	April 2015	June 2015
Pakistan	May 2015	July 2015
Somalia	October 2015	November 2015
Sudan	February 2015	June 2015
Tunisia	September 2014	September 2014
Yemen	November 2014	November 2015

Abbreviation: IPV, inactivated polio vaccine.

The global effort to introduce IPV in all countries has been complicated by major shortfalls in the production of IPV as well as demand for IPV for use in mass campaign supplemental immunization activities (SIAs). A global shortage of IPV has resulted in introduction delays in approximately 20 countries worldwide until 2017. At least 29 other countries are expected to experience national stockouts of IPV before being resupplied [12]. Among EMR countries, Egypt was the only country affected by the shortage, and as a result of the shortage will not be able to introduce IPV until 2017.

## FINANCIAL SUPPORT OF IPV INTRODUCTION

EMR countries greatly benefitted from financial support for the introduction of IPV from GPEI. Of the 12 EMR countries introducing IPV as part of the Endgame Plan, 6 were eligible for financial support from GPEI administered by Gavi, the Vaccine Alliance. However, Egypt and Iran, both middle-income countries that did not meet eligibility for support for IPV introduction, expressed concerns about financial constraints that would prevent them from introducing IPV according to the Endgame timelines. WHO EMRO and UNICEF MENA staff advocated to GPEI on behalf of both countries. As a result, Egypt received support for 1 year’s supply of IPV and for operational costs of vaccine introduction. Iran requested and received support for operational costs of introduction.

## TECHNICAL SUPPORT FOR IPV INTRODUCTION

Multiple sources of technical support facilitated IPV introductions in EMR countries. A dedicated session was incorporated in the yearly EMR EPI manager meetings in 2014 and 2015, where all documents and updates on IPV introduction were shared with the countries of the region. These sessions provided a platform for countries to voice their specific concerns and needs. Following training workshops in Atlanta and Nairobi in 2014, consultants and staff from the WHO and UNICEF EMR regional offices and from other GPEI partner organizations provided direct technical support to most of the target countries for a variety of activities including development of IPV introduction plans, financial support applications, operational readiness, cold chain assessments and upgrades, training of health workers, and logistical preparations for IPV introduction. In addition, an assessment of IPV introduction in Tunisia was conducted in 2014 in collaboration with Johns Hopkins University. The experience from Tunisia highlighted the motivation and enthusiasm of decision makers toward polio eradication, the highly developed national decision-making capacity and structure of the country’s National Immunization Technical Advisory Group, and the national government’s concerns for health equity which prompted it to create budgetary space for IPV introduction. The findings from this evaluation were circulated to other EMR countries to facilitate and to motivate additional IPV introductions, including a video that documented IPV introduction in Tunisia’s RI system [17]. Future efforts will focus on evaluations of IPV introduction and coverage assessments in target countries to assess outcomes of introduction efforts.

## REGISTRATION OF IPV AND BOPV

Licensing of IPV and bOPV in all countries were important prerequisites for the global switch from tOPV to bOPV, and could have been a serious bottleneck for the Endgame given the variability in the registration process and the lack of established infrastructure to fast-track registration and of developed regulatory authorities, particularly in resource-poor settings. To enable a smooth introduction of IPV and switch from tOPV to bOPV, registration of the appropriate IPV presentations by the end of 2014 and of bOPV by the end of 2015 was ideal. IPV and bOPV were available from multiple manufacturers. These vaccines had been licensed in their country of origin by a National Regulatory Authority (NRA) and then extensively reviewed and approved by the WHO prequalification program. Countries themselves have varying requirements to license vaccines and typically follow 1 of 3 types of regulatory pathways: accept WHO prequalified vaccines without requiring further registration, use a WHO-supported expedited review process to fast-track vaccine registration, or require full in-country review and registration of the vaccine. WHO EMRO staff mapped the regulatory requirements in each of the OPV using countries to determine licensure status for IPV and bOPV. In October 2014, WHO EMRO held several expedited review workshops for the joint evaluation of the marketing authorization files to be submitted by 2 IPV manufacturers that month. WHO EMRO also provided direct regulatory one-on-one support to selected countries, with a view to ensure that all countries had completed this important step by the end of 2014.

With regard to bOPV, the marketing authorization of the vaccine intended to be supplied was issued by the NRAs following the same regulatory pathway as IPV and was done smoothly because NRAs had experience with the registration and use of tOPV. WHO EMRO regularly followed up with the NRAs to ensure that appropriate bOPV products were registered by all countries before the tOPV-bOPV switch dates. Efforts are ongoing by WHO EMRO to ensure that NRAs from EMR countries complete the registration of bOPV vaccines from at least 2 manufacturers to prevent potential stockouts in case of shortage.

## SWITCH FROM TRIVALENT ORAL POLIOVIRUS VACCINE TO BIVALENT ORAL POLIOVIRUS VACCINE

Intense preparations for the switch and efforts by immunization managers and staff throughout the region resulted in all EMR countries ceasing official use of tOPV by 12 May 2016. Although 16 EMR countries reported that they had stopped use of tOPV by 1 May 2016, Egypt, Kuwait, Libya, Qatar, Syria, and the United Arab Emirates required a few more days to provide that confirmation to WHO EMRO. Egypt was the last country to report its cessation of tOPV use on 12 May. A tightly synchronized switch from tOPV to bOPV in which all countries switched within weeks of each other would have lower risks for subsequent cVDPV2 outbreaks than a loosely synchronized switch in which countries switched over many months [2, 9]. Preparations for the switch in the EMR began well in advance and included early advocacy and communications with country decision makers and senior management of EPI programs, workshops to provide platforms for discussion, dissemination of guidance, and training of consultants, catalytic funding support, and technical assistance.

After the May 2015 World Health Assembly resolution that urged all countries using OPV to prepare for an April 2016 global switch from tOPV to bOPV, staff from the WHO and UNICEF EMR regional offices were closely involved with the global planning activities on the switch, providing regional input on switch strategies, operational guidance, documents, and communications that were generated during late 2014 and early 2015. Adapting material from an early 2015 Atlanta workshop on the switch to meet EMR needs, the WHO and UNICEF EMR regional offices organized an August 2015 meeting in Cairo, Egypt, for immunization program managers and staff from countries throughout the region. This meeting involved a briefing of participants on the rationale and context for the switch, discussions of challenges in carrying out the switch, and possible ways of addressing those challenges. Countries drafted preliminary plans for conducting the switch during small facilitated group sessions. The WHO and UNICEF EMR regional offices later held a follow-up meeting in Amman, Jordan in March 2016 that allowed immunization program managers to review advanced plans for the switch and discuss how to resolve any outstanding problems with their preparations. An internet-based training session (“webinar”) organized by UNICEF MENA and supported by the Task Force for Global Health, in February 2016, also provided orientation and update to the switch and its component activities to hundreds of immunization staff members throughout the region.

Direct support to countries from WHO, UNICEF, and other GPEI partner organizations also facilitated the switch. A specific workshop on the switch was conducted in Pakistan with involvement of staff from all of Pakistan’s provinces. Staff visits to Tunisia, Djibouti, and Yemen were deemed by countries to be supportive to their programs for planning and implementing the switch. WHO EMRO and UNICEF MENA supported a specific session on the switch in Tunis to support the program in Libya, which has been under severe internal conflicts. The WHO and UNICEF EMR regional offices had routine communications with all countries to gather feedback, provide support, and track progress toward the switch.

Ultimately, the success of the switch in the EMR was a direct result of the motivation, engagement, and cooperation of immunization staff and decision makers across all national levels. While global and regional efforts were needed to jump-start the process and to provide the initial strategic planning, coordination, and guidance, particularly to facilitate the synchronization in April–May 2016, the switch was a set of national activities that were adapted from standardized global and regional guidance and tailored by countries to meet the local needs. Country ownership in developing plans, seeking funds, identifying priorities, lobbying leadership, conducting inventories, expediting bOPV registration, establishing committees with roles and responsibilities, training workers, developing monitoring and disposal strategies, and dealing and implementing creative local solutions, particularly in conflict situations, was ultimately the reason for the success of the switch in the EMR.

To ensure that no countries were prevented from completing the switch from tOPV to bOPV during the goal switch period of 17 April to 1 May because of lack of financial resources, the WHO and UNICEF regional offices worked with GPEI to secure financial assistance for countries when needed. Countries developed switch budgets and funding requests with support from the WHO and UNICEF regional offices, reflecting the total funds needed to conduct the switch, the proportion funded by the government and partners, and the funding gap requested from GPEI. The budgets were submitted to GPEI for approval. Ultimately, Afghanistan, Egypt, Jordan, Libya, Pakistan, Somalia, Sudan, Tunisia, and Yemen received catalytic financial support for the switch from GPEI; US$3.3 million were allocated through GPEI, including an additional increase of US$0.5 million to support Libya because of its critical situation. In addition, US$50 000 were provided by WHO EMRO to support Djibouti and Tunisia.

To maximize population immunity to type 2 poliovirus infections before the switch, 12 EMR countries collectively conducted 24 SIAs with tOPV between November 2015 and April 2016 (Table 2). Afghanistan, Djibouti, Egypt, Iraq, Jordan, Libya, Pakistan, Somalia, Syria, and Yemen each conducted at least 1 national immunization day (NID) campaign involving the entire country. In addition, Afghanistan, Egypt, Iraq, Lebanon, Sudan, and Syria each conducted at least 1 subnational immunization day (sNID) that targeted a portion of the country. The number of children targeted in these tOPV SIAs ranged from 6080 in a mop-up SIA held in Egypt in response to detection of an aVDPV2, to 35 717 767 in an NID held in Pakistan. Pakistan and Afghanistan also held IPV SIAs in selected areas as part of their efforts to end transmission of type 1 wild poliovirus; these SIAs also helped to boost immunity to type 2 polioviruses.

**Table 2.** Trivalent Oral Poliovirus Vaccine Supplemental Immunization Activities in Eastern Mediterranean Region, November 2015–April 2016

Country	No. of Supplemental Immunization Activities	Cumulative Target Population
Afghanistan	2 NIDs, 1 sNID	18 311 656
Djibouti	1 NID	129 578
Egypt	1 NID, 1 sNID, 1 Mop-up	13 432 335
Iraq	1 NID, 1 sNID	9 035 177
Jordan	1 NID	904 261
Lebanon	2 sNIDs	384 870
Libya	1 NID	Unknown
Pakistan	1 NID	35 717 767
Somalia	2 NIDs	3 916 470
Sudan	2 sNIDs	8 915 674
Syrian Arab Republic	1 NID, 1 sNID	3 726 471
Yemen	2 NIDs	10 981 274

Source: Global Polio Eradication Initiative Polio Information System, 4 October 2016.

Abbreviations: NID, National Immunization Day, sNID, Subnational Immunization Day.

All EMR countries conducted monitoring of the switch, drawing entirely on their own human resources for that monitoring. Monitors inspected cold chain stores and health facilities for the presence of bOPV and any residual tOPV withdrawn. Monitors also checked for the presence of IPV if a country had introduced IPV by the time of the switch. The monitors’ findings were reviewed by independent national switch validation committees, whose members were drawn from outside government. These committees in turn advised each country’s ministry of health on whether or not tOPV had been successfully withdrawn from the cold chain after the switch, or if any necessary corrective action was needed. Once a switch validation committee found that tOPV had been successfully withdrawn in its country, the ministry of health reported the findings to WHO EMRO. By 15 May, 2 weeks after the goal switch date, 10 EMR countries had reported that tOPV had been successfully withdrawn, and that total rose further to 17 by 23 May. By 30 June, all EMR countries except for Iraq and Libya had reported positive conclusions from their switch validation committees. By 30 September, Libya had also reported a positive conclusion from its switch validation committee, but Iraq indicated that it had not fully withdrawn tOPV as it retained a large stock of tOPV in its central cold store even though it had stopped use of tOPV.

## COUNTRIES’ SPECIFIC EXPERIENCE FOR THE SWITCH IN THE EASTERN MEDITERRANEAN REGION

The experiences of Egypt, Syria, Iraq, and Libya illustrate some of the challenges of carrying out the synchronized global switch from tOPV to bOPV within the goal time period of 17 April to 1 May. Egypt’s principal challenge related to the country not receiving expected supplies of IPV before the switch because of the global shortage of IPV.

As a result of the shortage, in early 2016 GPEI decided to include Egypt among the 20 countries that would not receive their initial supply of IPV until the fourth quarter of 2017. GPEI had prioritized supplying IPV first to Afghanistan and Pakistan because of their endemic wild poliovirus type 1 transmission; second to the other countries considered to be at highest risk for cVDPV2 because of their having cVDPV outbreaks since 2000 or estimated RI coverage of <80% for DTP3; third to SIAs conducted in response to polio outbreaks; and finally to countries considered to be at low risk for polio outbreaks. Even though Egypt had experienced cVDPV2 outbreaks in the 1980s and 1990s, it was considered to be at relatively low risk because it had not had a cVDPV outbreak since 2000 and had relatively high immunization coverage. However, in October 2015, SAGE had recommended that the globally synchronized switch in April 2016 proceed despite the global IPV shortage because the benefits of proceeding with the switch outweighed the risks from cVDPV2s related to delays in IPV introduction in the countries deemed to be at low risk for polio outbreaks [18]. In light of these developments, the Egyptian immunization program had to decide whether or not to follow SAGE’s October 2015 recommendations and proceed with the switch in April or May 2016 before introducing IPV.

Egyptian officials were wary about proceeding with the switch without IPV, and their concerns about the possibility of cVDPV2 outbreaks following the switch were exacerbated by the detection of multiple ambiguous VDPV2s (aVDPV2s), VDPV2s isolated only from a single acute flaccid paralysis case or from a single environmental sample and not linked to an individual with known immunodeficiency, in late 2015 and early 2016. After learning that GPEI would not be supplying it with IPV until 2017, Egypt tried to secure additional tOPV on the international market in April 2016 but found that all OPV manufacturers had ceased selling tOPV. However, at the same time, staff from WHO EMRO and WHO headquarters in Geneva worked to convince Egyptian officials to proceed with the switch by providing the rationale for GPEI’s allocation of IPV to countries as well as scientific evidence and policy support for proceeding with the switch in the absence of IPV. The rationale for proceeding with the switch despite the absence of IPV was further bolstered by the fact that Egypt’s population immunity to type 2 polioviruses had been increased, and the risk from cVDPV2s correspondingly decreased, by a national tOPV SIA in February 2016 as well as by a smaller subnational SIA with tOPV in December 2015. Ultimately, Egypt chose to use its last reserves of tOPV in an SIA in response to an aVDPV2 detected in environmental samples in the Sinai Peninsula in March 2016 and ceased tOPV use by 12 May 2016. Egypt already had a stockpile of bOPV in-country and was able to switch to bOPV immediately. After the completion of its switch monitoring, Egypt reported that monitors had visited its national vaccine cold store, 27 governorate cold stores, 281 district cold stores, and 454 health facilities and had found that all of them had withdrawn tOPV in the cold chain and switched to bOPV.

Syria faced a different set of challenges because it had introduced IPV in 2008 but continued to use tOPV. As of April 2016, it had not yet procured bOPV. At one point the Syrian Ministry of Health indicated that it would switch from tOPV to bOPV in September 2016, which could have posed a risk of transmitting type 2 polioviruses to countries that had ceased tOPV use [11]. However, after discussions with WHO EMRO and WHO headquarters, Syria agreed to switch from tOPV to bOPV in early May. Fortunately, Syria was able to purchase and quickly receive bOPV directly from a manufacturer, allowing tOPV use to stop on 1 May 2016. Following the completion of its switch monitoring, Syria reported on 22 May 2016 that monitors had visited its 3 national vaccine cold stores, 13 governorate cold stores, 67 district cold stores, and 357 health facilities and had found that although none of them had tOPV or bOPV in the cold chain, 93% of district cold stores and 60% of health facilities had IPV in stock.

Libya’s challenges during the switch related primarily to the difficulty of conducting the switch in a country with a sizable amount of civil disorder as well as weak communication and coordination between the various national and provincial governments. Libya was able to secure bOPV by April 2016 and officially switched from tOPV to bOPV by 1 May. Due to ongoing civil disorder, its immunization program had problems moving tOPV for disposal, and its monitors had difficulties visiting all of district cold stores and health facilities. Following the completion of its switch monitoring, Libya reported on 3 September 2016 that monitors had visited its 1 national vaccine cold store, 3 provincial cold stores, 25 district cold stores, and 231 health facilities and had found no tOPV vials in the cold chain.

Iraq faced difficulties during the switch with conducting all needed activities in the midst of civil disorder and deciding what to do with its excess tOPV after the switch. While Iraq officially switched from tOPV to bOPV by 30 April, it initially had difficulties in ensuring that the switch actually took place in all parts of the country. In addition, at the time of the switch Iraq considered retaining 0.97 million doses of tOPV that it had in its national cold store for use in RI or SIAs in case it ever had a shortage of bOPV. Discussions with senior staff from WHO EMRO and WHO headquarters highlighted the risks to the population of Iraq and other countries if tOPV were used after the switch. Monitoring of tOPV in Iraq’s cold chain was expected to be finalized once Iraq removes its remaining tOPV from its national cold store.

## CONCLUSIONS

Overall, the supporting effort to introduce IPV in all countries and the switch from tOPV to bOPV in the EMR and was very successful. The coordinated simultaneous withdrawal of tOPV and introduction of bOPV within and across the 22 EMR countries using OPV despite the many logistical and political challenges was a major accomplishment for the region’s immunization programs, as was the introduction of IPV in 11 countries between 2013 and 2016. That success indicates the importance of the multifaceted approach to reducing the risks associated with the switch, particularly in light of the challenges that arose in executing individual components of the risk reduction strategy. The tOPV SIAs in Egypt carried out in 2015 and 2016 in preparation for the switch provided additional population immunity that helped to mitigate any additional risks that accrued from the global IPV supply shortage preventing Egypt from introducing IPV in 2015. Conversely, the use of IPV in Syria helped provide children protection against polio caused by types 1 and 3 polioviruses when the introduction of bOPV was delayed. The careful monitoring of the switch at the local, national, and regional levels helped to alert WHO EMRO and UNICEF MENA to Iraq’s keeping tOPV in its national cold store after the switch, and to alert Iraq to the importance of not using the remaining tOPV. The long lead time for the switch also allowed lengthy, essential preparations for the switch, such as the registration and ordering of bOPV, to be completed in time. Multifaceted approaches to solving problems and addressing risks, flexibility in applying those approaches, and adequate time for preparation will be needed in future steps to eliminate the risks posed by use of OPV, including the eventual withdrawal of bOPV.

The introduction of IPV and synchronized switch from tOPV to bOPV in almost all EMR countries not using it in 2013 are also an indication of what the EMR EPI and GPEI can accomplish, particularly when supported with funding, technical assistance, and other resources. The cooperation shown among all EMR countries in an effort targeting polio regardless of national income levels or other differences can also serve as a model for efforts against other diseases, including measles and rubella.

## Notes

***Financial support.*** This work was supported by funds from the GPEI, WHO, UNICEF, and the CDC. M. P. was supported by the Bill and Melinda Gates Foundation (grant OPP1095024).

***Supplement sponsorship.*** This work is part of a supplement coordinated by the Task Force for Global Health with funding provided by The Bill and Melinda Gates Foundation and the Centers for Disease Control and Prevention.

***Potential conflicts of interest.*** All authors: No reported conflicts of interest. All authors have submitted the ICMJE Form for Disclosure of Potential Conflicts of Interest. Conflicts that the editors consider relevant to the content of the manuscript have been disclosed.
